# Dietary Zn proteinate with moderate chelation strength alleviates heat stress-induced intestinal barrier function damage by promoting expression of tight junction proteins via the A20/NF-κB p65/MMP-2 pathway in the jejunum of broilers

**DOI:** 10.1186/s40104-024-01075-8

**Published:** 2024-09-01

**Authors:** Yangyang Hu, Weiyun Zhang, Ke Yang, Xi Lin, Hsiao-Ching Liu, Jack Odle, Miles Todd See, Xiaoyan Cui, Tingting Li, Shengchen Wang, Xiudong Liao, Liyang Zhang, Sufen Li, Yun Hu, Xugang Luo

**Affiliations:** 1 Poultry Mineral Nutrition Laboratory, College of Animal Science and Technology, Yangzhou University, Yangzhou, 225000 China; 2 Hebei Normal University of Science and Technology, Qinhuangdao, 066004 China; 3 Department of Animal Science, North Carolina State University, Raleigh, NC 27695 USA; 4 Mineral Nutrition Research Division, State Key Laboratory of Animal Nutrition, Institute of Animal Science, Chinese Academy of Agricultural Sciences, Beijing, 100193 China

**Keywords:** A20/NF-κB p65/MMP-2 pathway, Broiler, Heat stress, Intestinal barrier function, Tight junction proteins, Zn

## Abstract

**Background:**

The aim of this study was to determine whether and how Zn proteinate with moderate chelation strength (Zn-Prot M) can alleviate heat stress (HS)-induced intestinal barrier function damage of broilers. A completely randomized design was used for comparatively testing the effects of Zn proteinate on HS and non-HS broilers. Under high temperature (HT), a 1 (Control, HT-CON) + 2 (Zn source) × 2 (added Zn level) factorial arrangement of treatments was used. The 2 added Zn sources were Zn-Prot M and Zn sulfate (ZnS), and the 2 added Zn levels were 30 and 60 mg/kg. Under normal temperature (NT), a CON group (NT-CON) and pair-fed group (NT-PF) were included.

**Results:**

The results showed that HS significantly reduced mRNA and protein expression levels of claudin-1, occludin, junctional adhesion molecule-A (JAMA), zonula occludens-1 (ZO-1) and zinc finger protein A20 (A20) in the jejunum, and HS also remarkably increased serum fluorescein isothiocyanate dextran (FITC-D), endotoxin and interleukin (IL)-1β contents, serum diamine oxidase (DAO) and matrix metalloproteinase (MMP)-2 activities, nuclear factor kappa-B (NF-κB) p65 mRNA expression level, and protein expression levels of NF-κB p65 and MMP-2 in the jejunum. However, dietary supplementation with Zn, especially organic Zn as Zn-Prot M at 60 mg/kg, significantly decreased serum FITC-D, endotoxin and IL-1β contents, serum DAO and MMP-2 activities, *NF-κB p65* mRNA expression level, and protein expression levels of NF-κB p65 and MMP-2 in the jejunum of HS broilers, and notably promoted mRNA and protein expression levels of claudin-1, ZO-1 and A20.

**Conclusions:**

Our results suggest that dietary Zn, especially 60 mg Zn/kg as Zn-Prot M, can alleviate HS-induced intestinal barrier function damage by promoting the expression of TJ proteins possibly via induction of A20-mediated suppression of the NF-κB p65/MMP-2 pathway in the jejunum of HS broilers.

## Background

As the global climate warms, animals suffer from more severe heat stress (HS) in the summer. The HS is one of the primary stressors that causes protein breakdown and ultimately results in aging and mortality [1]. The gastrointestinal tract is considered as the main target of HS [2]. Broilers are more susceptible to HS because they do not have sweat glands [3–5]. Previous studies have shown that HS damages the intestinal integrity and barrier function of broilers [6, 7], reducing health and growth performance and causing great economic losses in the poultry industry. Damage to the barrier function of the intestines is a primary factor leading to reduced growth rates and increased susceptibility to diseases [8].

In animals, intestinal barrier function of broilers is closely correlated to the expression levels of tight junction (TJ) proteins [9, 10]. Alhotan et al. [11] found that HS damages intestinal barrier function of broilers by reducing the mRNA expression levels of claudin-1, claudin-4, occludin and zonula occludens-1 (*ZO-1*). Deng et al. [12] reported that HS injures the barrier function of the small intestine by decreasing duodenal mRNA expression of occludin in broilers. Zhang et al. [13] showed that HS damages the intestinal barrier function of broilers by reducing the protein expression levels of claudin-1 and occludin. The above studies indicate that HS causes intestinal barrier dysfunction in broilers by decreasing the expression of TJ proteins. Heat stress-induced decrease in the expression of TJ proteins is associated with NF-κB activation in broilers [14], which in turn may promote the expression of matrix metalloproteinases 2 and 9 (MMPs 2 and 9) genes [15]. The MMPs are a class of endopeptidases that is responsible for degrading the TJ proteins, and only MMP-2 and MMP-9 are associated with the intestinal TJ proteins degradations [16, 17]. Therefore, HS may contribute to injuring small intestine barrier function in broilers possibly by suppressing TJ protein levels via activation of the NF-κB/MMP signaling pathway.

Food digestion and nutrient absorption are located mainly in the intestines [18]. The HS-induced intestinal barrier function damage causes digestive and absorption disorders as well as other diseases [19, 20]. Feed nutrition is one of the important measures to alleviate HS. Zinc (Zn) is an essential trace element for animals and human beings [21, 22]. Dietary Zn deficiency leads to decreased growth performance and feed utilization, impaired immune response and delayed wound healing [23–25]. Therefore, the addition of Zn to broiler diets to prevent Zn deficiency is required. The Zn, especially organic Zn, has potential benefits for alleviating HS-induced intestinal barrier function impairment in mammals [26] and in broilers [27–29]. The molecular mechanistic actions of inorganic Zn, have been investigated in other species [10, 30, 31]. Zinc oxide maintained the intestinal barrier function of weaning piglets by promoting the expression of TJ proteins [30]. Li et al. [10] reported that Zn oxide nanoparticles relieved HS-induced down-regulation of claudin and *ZO-1* expressions in bovine intestinal epithelial cells in vitro by inhibiting the NF-κB pathway. Yan et al. [31] found that the Zn finger protein A20 (A20) is involved in the regulation of *NF-κB* expression in rats. In addition, a series of studies with broilers in our laboratory has demonstrated that organic Zn as Zn proteinate with moderate chelation strength (Zn-Prot M) displays better Zn absorption and the highest Zn bioavailability compared to Zn sulfate and other organic Zn sources [32–35]. However, whether and how organic Zn-Prot M can alleviate HS-induced intestinal barrier function damage in broilers has not been reported.

The jejunum has been shown to be the most sensitive segment of the small intestine in broilers in response to Zn and HS [36–38]. We thus hypothesized that Zn, especially the organic form of Zn as Zn-Prot M, could alleviate the HS-induced intestinal barrier function damage in broilers by promoting the expression of TJ proteins via induction of A20-mediated suppression of NF-κB/MMP (A20/NF-κB/MMP) signaling pathway in the jejunum of broilers. The aim of the present study was to determine the effects of dietary added Zn source and Zn level on growth performance, intestinal barrier function, expression of jejunal TJ proteins, and A20/NF-κB/MMP signaling pathway genes of HS broilers.

## Materials and methods

### Animal ethics

The experiment was approved by the Animal Ethics Committee of Yangzhou University [approval number: SYXK (Su) 2021-0027].

### Experiment design and treatments

A completely randomized design was employed in this experiment. Under high temperature (HT, 9:00–17:00: 34 ± 1 °C, 8 h/d; 17:00–9:00: 28 ± 1 °C), a 1 (Control, HT-CON) + 2 [Zn source, ZnSO_4_·7H_2_O (ZnS) and Zn-Prot M (17.09% Zn and Q_f_ = 51.6 by analysis)] × 2 [added Zn level, 30 and 60 mg/kg] factorial arrangement of treatments was used. The two dietary added Zn levels were based on previous studies [27, 28]. Under normal temperature (NT, 23 ± 1 °C, 24 h/d), a CON group (NT-CON) and pair-fed (NT-PF) group (the feed intake restriction to the same as that for the HT-CON group) were set up. Thus, there were a total of 7 different treatments (NT-CON, NT-PF, HT-CON, HT-ZnS 30, HT-ZnS 60, HT-Zn-Prot M 30 and HT-Zn-Prot M 60). The experimental treatments are shown in Table 1.

**Table 1:** The arrangement of experimental treatments for 22–42-day-old broilers

Temperature	Added Zn source	Added Zn level, mg/kg	Analyzed Zn content, mg/kg
Normal temperature (NT, 23 ± 1 °C, 24 h/d; humidity: 55% ± 5%)	Zn sulfate (ZnS) (Control group, NT-CON)	40^a^	64.36
	ZnS (Pair-fed group, NT-PF^c^)	40^a^	64.36
High temperature (HT, 9:00–17:00: 34 ± 1 °C, 8 h/d; 17:00–9:00: 28 ± 1 °C; humidity: 55% ± 5%)	ZnS (Control group, HT-CON)	40^a^	64.36
	ZnS (HT-ZnS)	30^b^	91.10
		60^b^	120.43
	Zn proteinate with moderate chelation strength (HT-Zn-Prot M)	30^b^	91.74
		60^b^	121.00

^a^Supplemental Zn level based on dietary Zn requirement

^b^Added Zn levels to the above control group diet

^c^The feed intake of birds in the NT-PF group was restricted to the same as that of those in the HT-CON group

### Animals and diets

A total of 1,150 one-day-old Arbor Acres (AA) male broilers with an average body weight (41.3 ± 0.1 g) were purchased from Jiangsu Jinghai Poultry Group Co., Ltd., China. All chickens were fed ad libitum with the same corn-soybean meal complete diet formulated based on the NRC [39] and Feeding standard of chicken in China [40] during d 1–21 (Table 2). At d 22 of age, 784 chickens were weighed, selected, and randomly allotted to 1 of 7 treatments with 7 replicates and 16 birds per replicate. The 16 chickens were placed in two adjacent stainless steel cages equipped with stainless steel feeders and waterers with 8 chickens per cage (90 cm × 70 cm × 45 cm). The two adjacent cages were regarded as one replicate unit. The broilers were maintained on a 24-h constant light schedule and handled in accordance with the Arbor Acres Broiler Management Guide [41], and allowed ad libitum access to diets (except for the NT-PF group chickens) and to tap water containing no detectable Zn. The room temperature for the NT-CON and NT-PF groups was maintained at 23 ± 1 °C daily, whereas the room temperature for the HT-CON, HT-ZnS-30, HT-ZnS-60, HT-Zn-Prot M-30 and HT-Zn-Prot M-60 groups was set as 34 ± 1 °C, 8 h/d, and the temperature for the remaining time was set as 28 ± 1 °C. The relative humidity under either NT or HT was maintained at 55% ± 5%. As the broilers in the HT-CON group had lower feed intake than those in the NT-CON group, to eliminate the potential effect of the reduced feed intake in the HT-CON group, the broilers in the NT-PF group were pair-fed the same amount of feed as those in the HT-CON group consumed on the previous day. Dead birds were recorded daily, and body weight and feed intake were measured per replicate unit on d 28, 35 and 42 to calculate the average daily feed intake (ADFI), average daily gain (ADG), feed-to-gain ratio (F/G), and mortality during d 22 to 42.

**Table 2:** Composition and nutrient levels of the broiler complete diets (as-fed basis)

Item	D 1–21	D 22–42
Ingredient, %		
Corn	54.75	58.78
Soybean meal	36.31	32.56
Soybean oil	4.95	5.15
CaHPO_4_^a^	1.87	1.67
CaCO_3_^a^	1.20	1.09
NaCl^a^	0.30	0.30
DL-Methionine^b^	0.31	0.15
Micronutrients^c^	0.31	0.20
Corn starch^d^	0.00	0.10
Total	100.00	100.00
Nutrient levels, %		
Metabolizable Energy, kcal/kg	3,045	3,102
Crude protein^f^	21.44	20.08
Lysine^e^	1.10	1.01
Methionine^e^	0.61	0.44
L-Threonine^e^	0.80	0.75
Tryptophan^e^	0.24	0.22
Methionine + Cysteine^e^	0.91	0.72
Ca^f^	1.01	0.92
Non-phytate P^e^	0.45	0.40
Zn, mg/kg^f^	84.40	64.36

^a^Reagent grade

^b^Feed grade

^c^Provided per kilogram of diet for d 1 to 21: Vitamin A 12,000 IU, Vitamin D_3_ 4,500 IU, Vitamin E 33 IU, Vitamin K_3_ 3 mg, Vitamin B_1_ (thiamin) 3 mg, Vitamin B_2_ (riboflavin) 9.6 mg, Vitamin B_6_ 4.5 mg, Vitamin B_12_ 0.03 mg, Pantothenic acid calcium 15 mg, Niacin 54 mg; Folic acid 1.5 mg, Biotin 0.15 mg; Choline 700 mg, Cu (CuSO_4_·5H_2_O) 6 mg, Fe (FeSO_4_·7H_2_O) 40 mg, Zn (ZnSO_4_·7H_2_O) 60 mg, Mn (MnSO_4_·H_2_O) 110 mg, Se (Na_2_SeO_3_) 0.35 mg, I (Ca(IO_3_)_2_·H_2_O) 0.35 mg; and for d 22 to 42: Vitamin A 8,000 IU, Vitamin D_3_ 3,000 IU, Vitamin E 22 IU, Vitamin K_3_ 2 mg, Vitamin B_1_ (thiamin) 2 mg, Vitamin B_2_ (riboflavin) 6.4 mg, Vitamin B_6_ 3 mg, Vitamin B_12_ 0.02 mg, Pantothenic acid calcium 10 mg, Niacin 36 mg; Folic acid 1.0 mg, Biotin 0.10 mg; Choline 500 mg, Cu (CuSO_4_·5H_2_O) 6 mg, Fe (FeSO_4_·7H_2_O) 30 mg, Zn (ZnSO_4_·7H_2_O) 40 mg, Mn (MnSO_4_·H_2_O) 80 mg, Se (Na_2_SeO_3_) 0.35 mg, I (Ca(IO_3_)_2_·H_2_O) 0.35 mg

^d^ZnSO_4_·7H_2_O and Zn proteinate with moderate chelation strength added in place of the equivalent weight of corn starch to produce treatment diets as shown in Table 1

^e^Calculated values

^f^Values determined by analysis, and each value is based on triplicate determinations

The corn-soybean meal complete diets were formulated to meet or exceed the requirements of broilers for all nutrients as recommended by the NRC [39] and Feeding standard of chicken in China [40] (Table 2). The supplemental levels of Zn as ZnS in the above basal complete diets as shown in Table 2, were based on the recent research results on dietary Zn requirements of broilers fed the conventional corn-soybean meal diets from 1 to 21 days of age or 22 to 42 days of age from our laboratory [42, 43]. During d 22–42, a single batch of the complete diet (CON) was mixed and then divided into 7 aliquots according to the experimental treatments. Either ZnS or Zn-Prot M was added to the above CON diet to formulate the respective treatment diets. The ZnS was reagent grade (Sinopharm Chemical Reagent Co. Ltd., Shanghai, China) and contained 22.49% Zn on a basis of analysis (purity > 99.5%). The Zn-Prot M was the same as that used in the study of Hu et al. [35], which was provided by a special commercial company and contained 17.09% Zn with the chelation strength (Q_f_ value) of 51.6 on a basis of analysis. The Q_f_ value of 51.6 is categorized as a moderate chelation strength based on the classification of Holwerda et al. [44]. Lysine and methionine levels in the control diet or the diet supplemented with the ZnS were balanced by adding synthetic lysine-HCl and DL-methionine based on supplemental amounts of lysine and methionine from the Zn-Prot M. The analyzed Zn contents in all treatment diets are shown in Table 1.

### Sample collection and preparation

Diet samples were collected and analyzed for calcium, Zn, or crude protein contents. The tap water was collected for analysis of Zn content. At 28, 35 and 42 days of age, 3 chickens from each replicate unit were selected based on the average body weight of the cage unit and then anesthetized by intraperitoneal injection of propofol (20 mg/kg body weight). As the biomarkers from the hepatic portal vein blood better reflect the intestinal barrier function and health status of animals, blood samples were collected from the hepatic portal vein of each bird, and serum was separated and stored at –20 °C for analyses of endotoxin, IL-1β and IL-6 contents and diamine oxidase (DAO) activity. The chickens were euthanized by decapitation after taking blood, and the jejunum was separated and rinsed with ice-cold saline solution. The jejunal mucosa were scraped with a sterilized microscope slide and then placed in sterilized 1.5-mL centrifuge tubes, immediately frozen in liquid nitrogen and stored at –80 °C for analyses of mRNA and protein expression levels. In addition, another chicken was selected from each replicate unit based on the average body weight of the cage unit, and subjected to fluorescein isothiocyanate dextran (FITC-D, catalog number FD4; Merck, Darmstadt, DEU) oral perfusion in a dose of 8.32 mg/kg body mass [45]. At 1 h after perfusion, blood samples were collected from the hepatic portal vein, and centrifuged at 3,000 r/min and 4 °C to obtain serum samples for analysis of FITC-D content. All samples from 3 birds in each replicate unit were pooled into 1 sample before analyses.

### Analyses of dietary crude protein, calcium and Zn contents, serum indices and jejunal MMP-2 activity

The crude protein content in the diets was determined using the Kjeldahl method [46]. The 5110 inductively coupled plasma optical emission spectrometry (Agilent Technologies, Mulgrave, Australia) was used to measure the calcium content in the diets, and Zn content in the diets and tap water as described previously [47]. Commercial kits were used to detect serum DAO activity (catalog number BC1285; Solarbio, Beijing, China), endotoxin (catalog number EC80545S; Bioendo, Xiamen, China), IL-1β (catalog number SEKCN-0153; Solarbio, Beijing, China) and IL-6 (catalog number SEKCN-0161; Solarbio, Beijing, China) contents, and jejunal MMP-2 activity (catalog number SEKCN-0022; Solarbio, Beijing, China). Serum FITC-D was measured by a multifunctional microplate reader as previously described [45].

### Quantitative real-time PCR

Total RNA was extracted from 50 mg jejunum using the TRIzol kit (catalog number 15596018CN; Invitrogen, Carlsbad, California, USA). A reverse transcription kit (catalog number R223-01; Vazyme, Nanjing, China) was used to reverse transcribe the RNA into cDNA, and quantitative real-time PCR was performed using SYBR Green (catalog number Q111-02; Vazyme, Nanjing, China). All primers (Table 3) were synthesized by Tsingke Biotech (Beijing, China). β-Actin and glyceraldehyde-3-phosphate dehydrogenase (GAPDH) were used as reference genes. The data were analyzed using the 2^−ΔΔCt^ relative expression method [48].

**Table 3:** Primer sequences for real-time PCR amplification

Genes	GenBank ID	Primer sequences	Product length, bp
Claudin-1	AY750897.1	F: 5'-CATACTCCTGGGTCTGGTTGGT-3'	100
		R: 5'-GACAGCCATCCGCATCTTCT-3'	
Occludin	NM_205128.1	F: 5'-GCAGATGTCCAGCGGTTACT-3'	150
		R: 5'-ATGACGATGAGGAACCCACA-3'	
*ZO-1*	XM_015278975	F: 5'-CTTCAGGTGTTTCTCTTCCTCCTC-3'	131
		R: 5'-CTGTGGTTTCATGGCTGGATC-3'	
*JAMA*	EF102433	F: 5'-CAGACCCCTACAAGAACCGC-3'	149
		R: 5'-CACCTGGACGATGAGGTTGA-3'	
*A20*	XR_005848478.2	F:5'-AAGAAGCCAGAAGGACAGAAGAAC-3'	83
		R: 5'-CAGTGCTGCTCGCTGTAGATC-3'	
*NF-κB p65*	NM_001012887.2	F: 5'-AAGATCTGGTGGTGTGCCTG-3'	137
		R: 5'-AGTGGAACCTTTCGCGGATT -3'	
*MMP-2*	NM_204420.3	F: 5'-CGATGCTGTCTACGAGTCCC-3'	96
		R: 5'-TAGCCCCTATCCAGGTTGCT-3'	
*MMP-9*	NM_204667.2	F: 5'-TCACGTACCGGGTGATGAAC-3'	288
		R: 5'-TCACGTACCGGGTGATGAAC-3'	
β-Actin	NM_205518.1	F: 5'- CAGCCATCTTTCTTGGGTAT-3'	169
		R: 5'- CTGTGATCTCCTTCTGCATCC-3'	
*GAPDH*	NM_204305.1	F: 5'-CTTTGGCATTGTGGAGGGTC-3'	128
		R: 5'-ACGCTGGGATGATGTTCTGG-3'	

*ZO-1* Zonula occludens-1, *JAMA* Junctional adhesion molecule-A, *A20* Zn finger protein A20, *NF-κB* Nuclear factor kappa-B, *MMP* Matrix metalloproteinase, *GAPDH* Glyceraldehyde-3-phosphate dehydrogenase, *F* Forward, *R* Reverse

### Total protein extraction and Western blotting

Frozen jejunal mucosa samples were homogenized in ice-cold RIPA lysis buffer (catalog number R0010; Solarbio, Beijing, China) as previously described [49]. The protein concentration was determined by BCA protein assessment kit (catalog number 23225; ThermoFisher, Waltham, Massachusetts, USA). Proteins were separated by SDS-PAGE, and transferred onto nitrocellulose (NC) membranes. The NC membrane was blocked at room temperature for 1.5 h using 5% BSA. After that, it was incubated overnight at 4 °C with primary antibodies [claudin-1 (catalog number WL03073; Wanlei, Shenyang, China; 1:1,000), occludin (catalog number 331500; Invitrogen, Carlsbad, California, USA; 1:1,000), ZO-1 (catalog number 617300; Life technologies, Carlsbad, California, USA; 1:250), JAMA (catalog number A1241; ABclonal, Wuhan, China; 1:1,000), A20 (catalog number WL00820; Wanlei, Shenyang, China; 1:500), NF-κB p65 (catalog number 10745-1-AP; Proteintech, Wuhan, China; 1:3,000), MMP-2 (catalog number bs-0412R; Bioss, Beijing, China; 1:1,000), MMP-9 (catalog number bs-4593R; Bioss, Beijing, China; 1:1,000), Lamin A/C (catalog number BS1446; Bioworld, Nanjing, China; 1:1,000) and β-actin (catalog number HX1827; Huaxingbio, Beijing, China; 1:5,000)]. On the following day, Tris-Buffered Saline and Tween 20 (TBST) were used to wash the membranes and then they were incubated with secondary antibody. The protein bands were visualized using the chemiluminescence (ECL) detection kit (catalog number 180-5001; Tanon, Beijing, China). Quantification of protein bands was performed with Tanon GIS 1D software (Tanon, Beijing, China). Protein quantification was performed using β-actin as the cytoplasmic internal reference and Lamin A/C as the nuclear internal reference.

### Statistical analyses

All data were analyzed statistically using SAS software (version 9.4, 2013). The data obtained from NT-CON, NT-PF and HT-CON groups were analyzed using the GLM procedure through one-way ANOVA. A single degree of freedom contrast was used to test the differences between all supplemental Zn treatments and the HT-CON. The data excluding the HT-CON under HT were analyzed by two-way ANOVA using the GLM procedure, and the statistical model included Zn source, added Zn level and their interaction. Differences among means were tested by the least significant difference (LSD) method. The replicate cage unit was the experimental unit and the statistical significance was set at *P* < 0.05.

## Results

### Growth performance and mortality

**Fig. 1:**
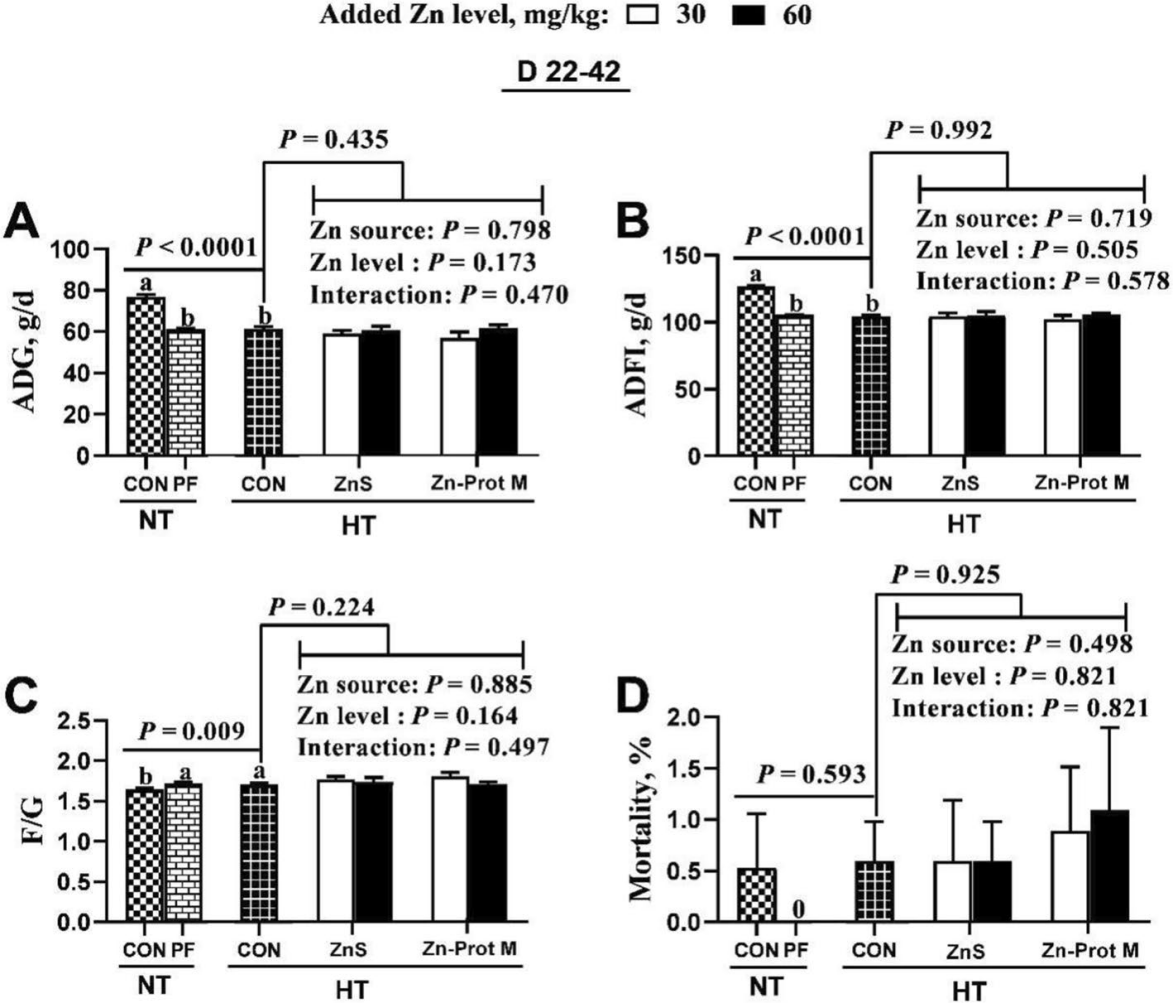
Effects of dietary added Zn source and level on growth performance and mortality of HS broilers from 22 to 42 days of age. **A** ADFI. **B** ADG. **C** F/G. **D** Mortality. ZnS: Zn sulfate. Zn-Prot M: Zn proteinate with moderate chelation strength (Q_f_  = 51.6). NT: normal temperature, 23 ± 1 °C, 24 h/d. HT: high temperature, 9:00–17:00: 34 ± 1 °C, 8 h/d, and 17:00–9:00: 28 ± 1 °C. ADFI: average daily feed intake. ADG: average daily gain. F/G: feed to gain ratio. CON: control group. PF: pair-fed group, and the feed intake of PF birds was restricted to match that of those in the HT-CON group. Different letters indicate significant differences among treatments (*P* < 0.05). Data are mean ± SE (*n* = 5–7)

As shown in Fig. 1, the treatment groups NT-CON, NT-PF and HT-CON did not affect mortality, but influenced (*P* < 0.01) ADG, ADFI and F/G of broilers during d 22–42. Compared with the NT-CON, HT-CON and NT-PF groups exhibited decreased (*P* < 0. 05) ADG and ADFI, but increased (*P* < 0.05) F/G with no differences between HT-CON and NT-PF groups. Compared with the HT-CON, all supplemental Zn treatments under HT had no effect on the growth performance indices (ADG, ADFI and F/G) and mortality of broilers from 22 to 42 days of age under HT. The Zn source, added Zn level and their interaction had no effects on the growth performance indices and mortality of broilers from 22 to 42 days of age under HT.

### FITC-D, endotoxin and interleukin contents and DAO activity in serum, and jejunal MMP-2 activity

**Fig. 2:**
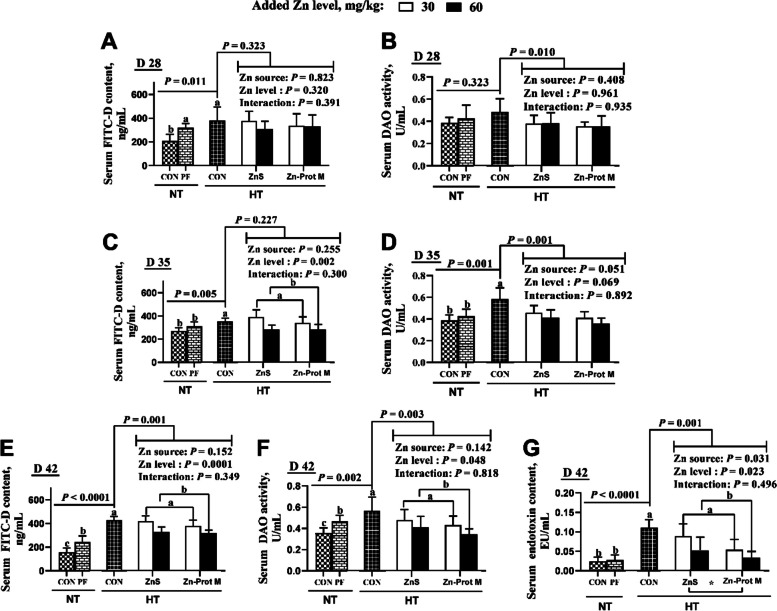
Effects of dietary added Zn source and level on serum FITC-D contents and DAO activities of HS broilers on d 28, 35 and 42 and serum endotoxin content of HS broilers on d 42. **A** Serum FITC-D content of broilers on d 28. **B** Serum DAO activity of broilers on d 28. **C** Serum FITC-D content of broilers on d 35. **D** Serum DAO activity of broilers on d 35. **E** Serum FITC-D content of broilers on d 42. **F** Serum DAO activity of broilers on d 42. **G** Serum endotoxin content of broilers on d 42. ZnS: Zn sulfate. Zn-Prot M: Zn proteinate with moderate chelation strength (Q_f_  = 51.6). NT: normal temperature, 23 ± 1 °C, 24 h/d. HT: high temperature, 9:00–17:00: 34 ± 1 °C, 8 h/d, and 17:00–9:00: 28 ± 1 °C. CON: control group. PF: pair-fed group, and the feed intake of PF birds was restricted to match that of those in the HT-CON group. FITC-D: fluorescein isothiocyanate dextran. DAO: diamine oxidase. Different letters indicate significant differences among treatments (*P* < 0.05). ^*^P < 0.05. Data are mean ± SE (*n* = 4–7)

As shown in Fig. 2, the treatment groups NT-CON, NT-PF and HT-CON did not affect serum DAO activity of broilers on d 28, but influenced (*P* < 0.04) serum FITC-D contents and DAO activities on d 35 and 42, and serum FITC-D content on d 28, and serum endotoxin content on d 42. Compared with the NT-CON, HT-CON and NT-PF increased (*P* < 0.05) serum FITC-D contents of broilers on d 28 and 42, and serum DAO activity on d 42. Compared with the NT-PF, HT-CON increased (*P* < 0.05) serum FITC-D content and DAO activities of broilers on d 35 and 42, and serum endotoxin content on d 42. Compared with the HT-CON, all supplemental Zn treatments under HT decreased (*P* < 0.05) serum DAO activities of broilers on d 28, 35 and 42, and serum FITC-D and endotoxin contents of broilers on d 42. Under HT, Zn source affected (*P* < 0.05) serum endotoxin content of broilers on d 42. Added Zn level affected (*P* < 0.05) serum FITC-D and endotoxin contents and DAO activity of broilers on d 42, and serum FITC-D content on d 35. There were no interactions between Zn source and added Zn level in all of the above indices. Compared with the ZnS, Zn-Prot M decreased (*P* < 0.05) serum endotoxin content in broilers on d 42. Compared with the 30 mg/kg of supplemental Zn, 60 mg/kg decreased (*P* < 0.05) serum FITC-D and endotoxin contents and DAO activity of broilers on d 42, and serum FITC-D content on d 35.

**Fig. 3:**
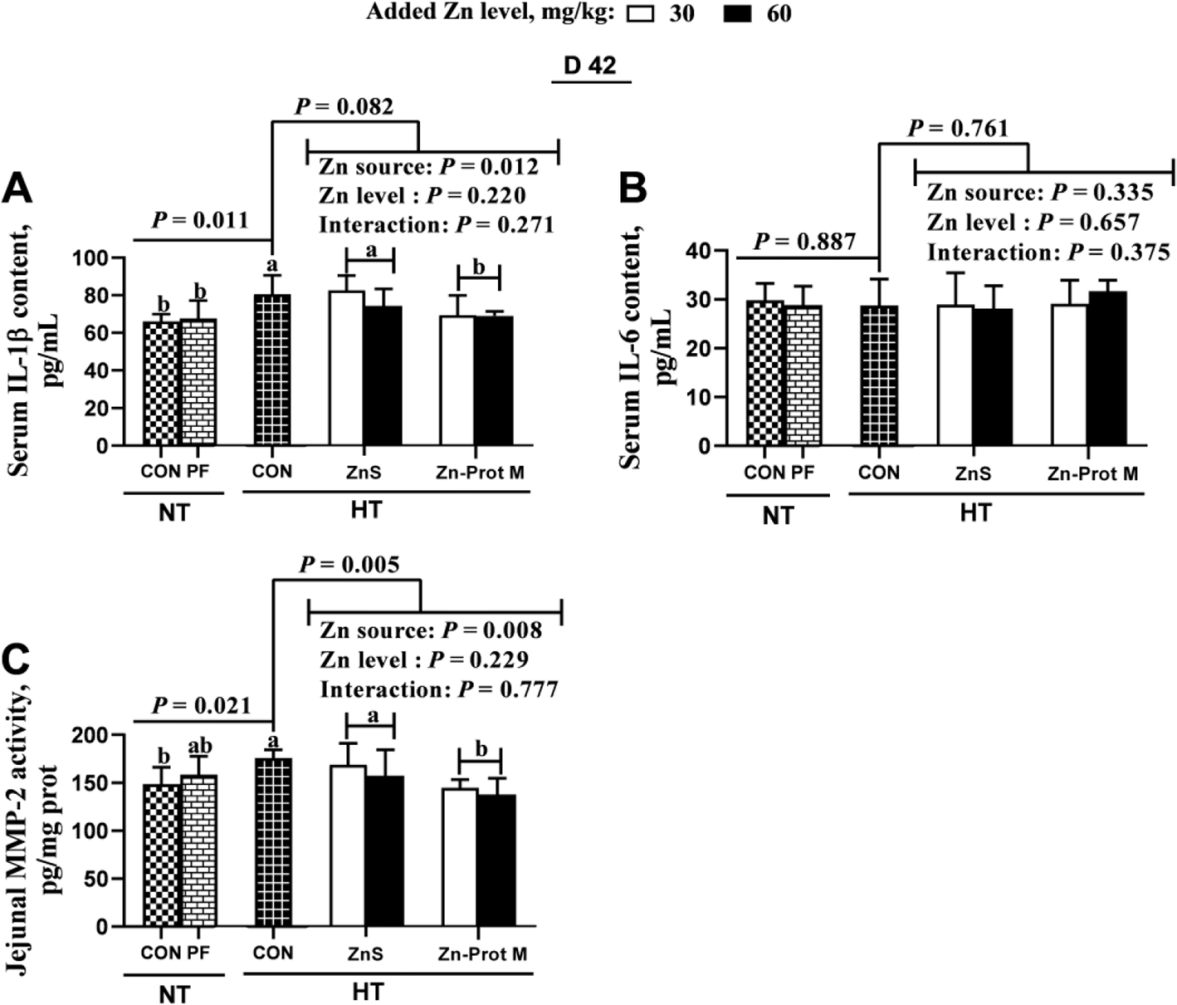
Effects of dietary added Zn source and level on serum IL-1β and IL-6 contents and jejunal MMP-2 activity of HS broilers on d 42. **A** Serum IL-1β content. **B** Serum IL-6 content. **C** Jejunal MMP-2 activity. ZnS: Zn sulfate. Zn-Prot M: Zn proteinate with moderate chelation strength (Q_f_ = 51.6). NT: normal temperature, 23 ± 1 °C, 24 h/d. HT: high temperature, 9:00–17:00: 34 ± 1 °C, 8 h/d, and 17:00–9:00: 28 ± 1 °C. CON: control group. PF: pair-fed group, and the feed intake of PF birds was restricted to match that of those in the HT-CON group. IL, interleukin. MMP, matrix metalloproteinase. Different letters indicate significant differences among treatments (*P* < 0.05). Data are mean ± SE (*n* = 5–7)

As shown in Fig. 3, the treatment groups NT-CON, NT-PF and HT-CON had no effect on serum IL-6 content, but influenced (*P* < 0.05) serum IL-1β content and jejunal MMP-2 activity of broilers on d 42. Compared with the NT-CON, HT-CON increased (*P* < 0.05) serum IL-1β content and jejunal MMP-2 activity. Compared with the NT-PF, HT-CON increased (*P* < 0.05) serum IL-1β, but did not change jejunal MMP-2 activity. Compared with the HT-CON, all supplemental Zn treatments under HT decreased (*P* < 0.01) jejunal MMP-2 activity, and had no effect on serum IL-1β and IL-6 contents. Under HT, Zn source did not affect serum IL-6 content, but affected (*P* < 0.05) serum IL-1β content and jejunal MMP-2 activity. Added Zn level and its interaction with Zn source did not affect serum IL-1β and IL-6 contents, and jejunal MMP-2 activity. Compared with the ZnS, Zn-Prot M decreased (*P* < 0.05) serum IL-1β content and jejunal MMP-2 activity.

### mRNA expression levels

**Fig. 4:**
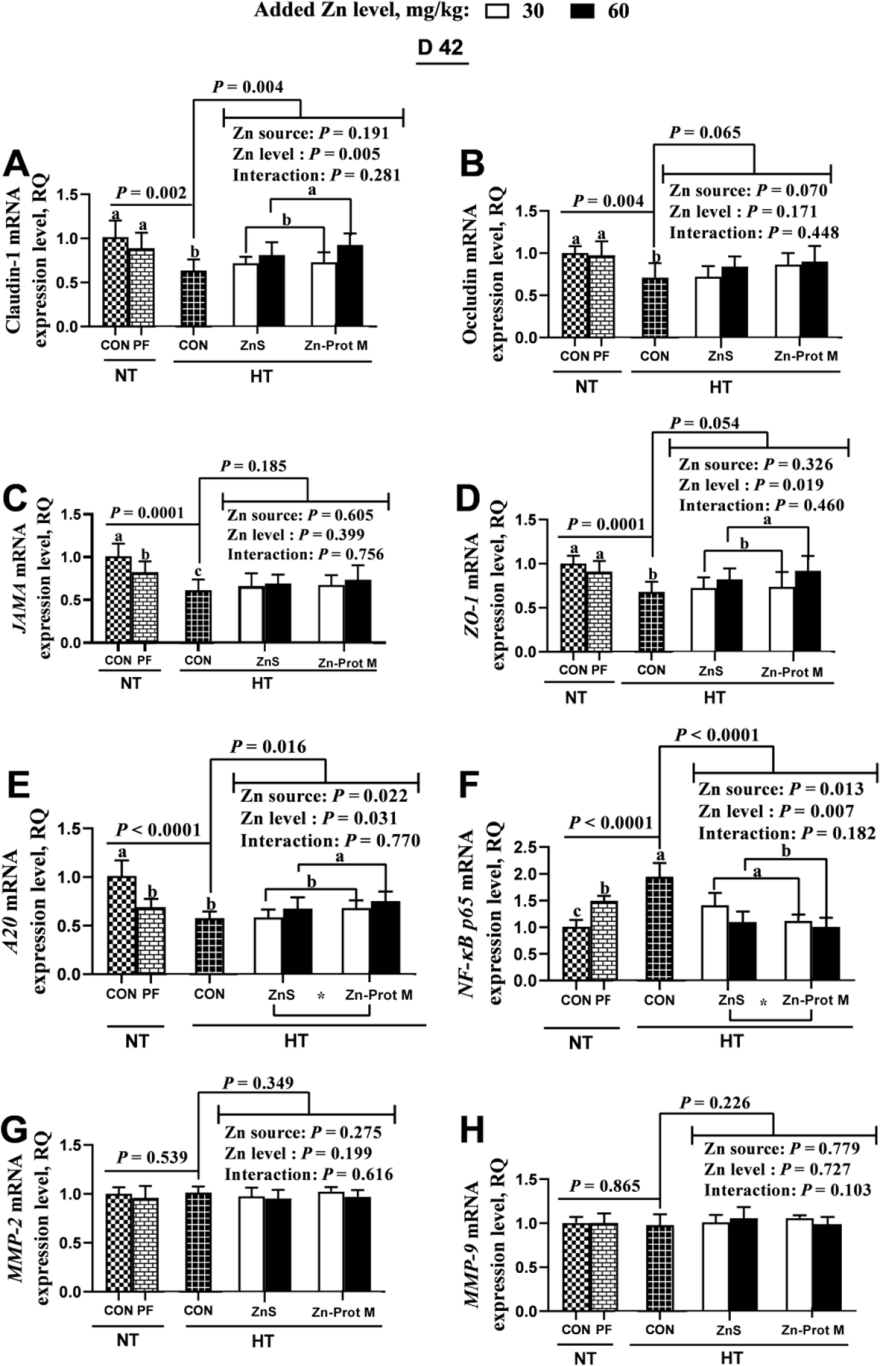
Effects of dietary added Zn source and level on mRNA expression levels of tight junction proteins and target proteins in the related signaling pathway in the jejunum of HS broilers at 42 days of age. **A–D** mRNA expression levels of related tight junction proteins (claudin-1, occludin, *JAMA* and *ZO-1*). **E–H** mRNA expression levels of target proteins (*A20*, *NF-ΚB p65*, *MMP-2* and *MMP-9*) in A20/NF-κB p65/MMP-2 signaling pathway. ZnS: Zn sulfate. Zn-Prot M: Zn proteinate with moderate chelation strength (Q_f_ = 51.6). NT: normal temperature, 23 ± 1 °C, 24 h/d. HT: high temperature, 9:00–17:00: 34 ± 1 °C, 8 h/d, and 17:00–9:00: 28 ± 1 °C. CON: control group. PF: pair-fed group, and the feed intake of PF birds was restricted to match that of those in the HT-CON group. *ZO-1*, zonula occludens-1; *JAMA*, junctional adhesion molecule-A; *MMP*, matrix metalloproteinase; *A20*, Zn finger protein A20; *NF-κB*, nuclear factor kappa-B. Values of mRNA abundance levels of target genes were calculated as the relative quantities (RQ) of claudin-1, occludin, *JAM-A*, *ZO-1*, *MMP2*, *MMP9*, *A20* or N*F-κB p65* mRNA to the geometric mean of internal reference genes *β-actin* and *GAPDH* mRNA using 2^−△△^^Ct^. Different letters indicate significant differences among treatments (*P* < 0.05). ^*^P < 0.05. Data are mean ± SE (*n* = 5–7)

As shown in Fig. 4, the treatment groups NT-CON, NT-PF and HT-CON had no effect on mRNA expression levels of *MMP-2* and *MMP-9*, but influenced (*P* < 0.01) mRNA expression levels of claudin-1, occludin, *JAMA*, *ZO-1*, *A20* and *NF-κB p65* in the jejunum of broilers on d 42. Compared with the NT-CON, either NT-PF or HT-CON decreased (*P* < 0.01) mRNA expression levels of *JAMA* and *A20,* but increased (*P* < 0.0001) *NF-κB p65* mRNA expression*.* Compared with the NT-PF, HT-CON decreased (*P* < 0.05) mRNA expression levels of claudin-1, occludin, *JAMA* and *ZO-1*, and increased (*P* < 0.05) *NF-κB p65* mRNA expression. Compared with the HT-CON, all supplemental Zn treatments under HT increased (*P* < 0.05) claudin-1 and *A20* mRNA expression levels, and decreased (*P* < 0.0001) *NF-κB p65* mRNA expression. Under HT, Zn source did not affect mRNA expression levels of claudin-1, occludin, *JAMA*, *ZO-1*, *MMP-2* and *MMP-9*, but influenced (*P* < 0.05) mRNA expression levels of *A20* and *NF-κB p65.* Added Zn level did not affect mRNA expression levels of occludin, *JAMA, MMP-2* and *MMP-9*, but influenced (*P* < 0.05) mRNA expression levels of claudin-1*, ZO-1, A20* and *NF-κB p65.* No interactions between Zn source and added Zn level were detected on mRNA expression levels for any of the above-mentioned genes. Compared with the ZnS, Zn-Prot M increased (*P* < 0.05) *A20* mRNA expression and decreased (*P* < 0.05) *NF-κB p65* mRNA expression. Compared with the 30 mg/kg of supplemental Zn, 60 mg/kg increased (*P* < 0.05) mRNA expression levels of claudin-1, *ZO-1* and *A20*, and decreased (*P* < 0.01) *NF-κB p65* mRNA expression.

### Protein expression levels

**Fig. 5:**
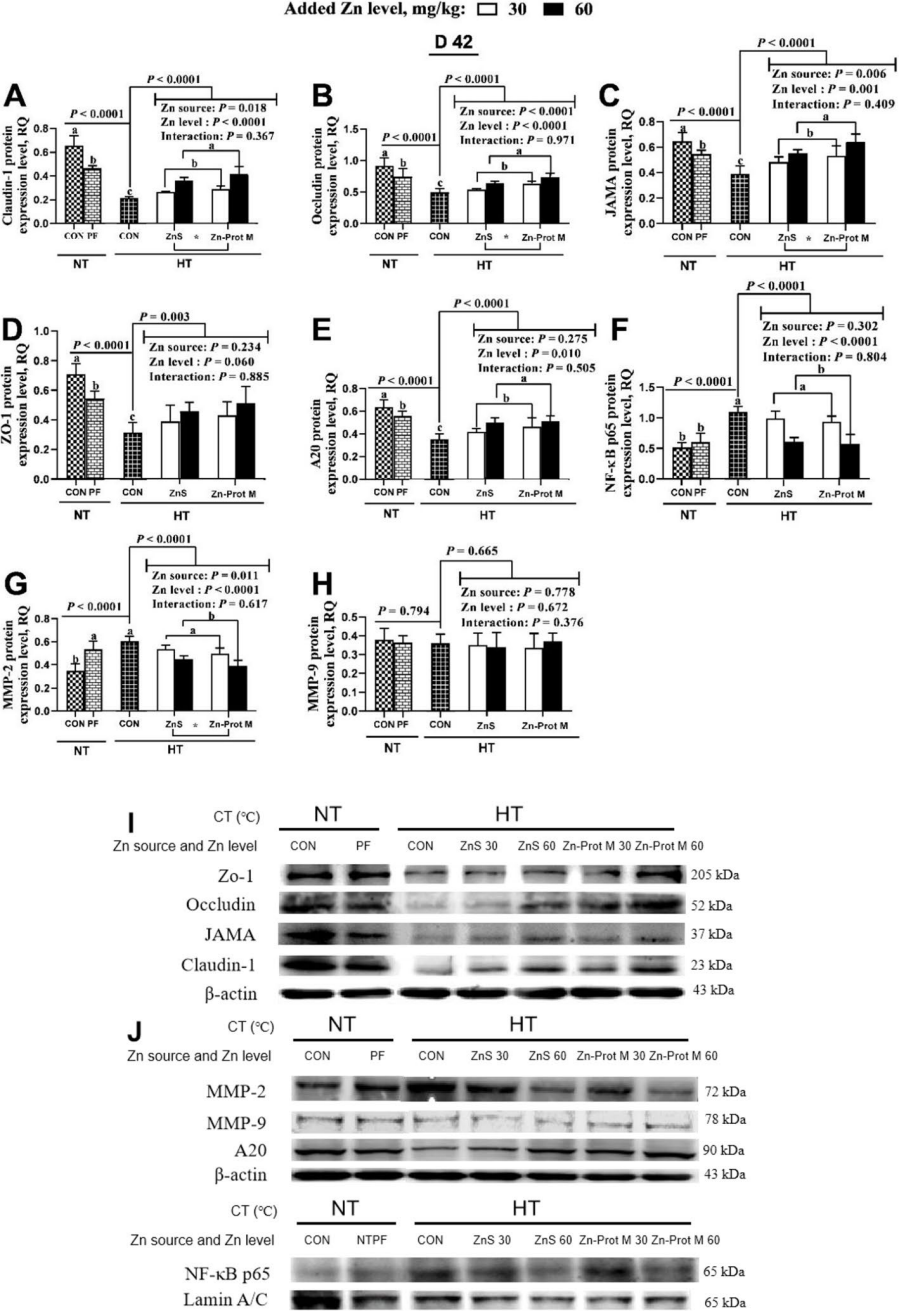
Effects of dietary added Zn source and level on protein expression levels of tight junction proteins and target proteins in the related signaling pathway in the jejunum of HS broilers at 42 days of age. **A–D** Protein expression levels of related tight junction proteins (claudin-1, occludin, JAMA and ZO-1). **E–H** Protein expression levels of target proteins (A20, NF-ΚB p65, MMP-2 and MMP-9) in A20/NF-κB p65/MMP-2 signaling pathway. **I** Representative immunoblots of related tight junction proteins (claudin-1, occludin, JAMA and ZO-1). **J** Representative immunoblots of target proteins (A20, NF-ΚB p65, MMP-2 and MMP-9) in A20/NF-κB p65/MMP-2 signaling pathway. ZnS: Zn sulfate. Zn-Prot M: Zn proteinate with moderate chelation strength (Q_f_  = 51.6). NT: normal temperature, 23 ± 1℃, 24 h/d. HT: high temperature, 9:00–17:00: 34 ± 1 °C, 8 h/d, and 17:00–9:00: 28 ± 1 °C. CON: control group. PF: pair-fed group, and the feed intake of PF birds was restricted to match that of those in the HT-CON group. ZO-1, zonula occludens-1; JAMA, junctional adhesion molecule-A. MMP, matrix metalloproteinase; A20, Zn finger protein A20; NF-κB, nuclear factor kappa-B. Values of target protein expression levels were expressed by the relative quantities (RQ) of target protein band intensity to the internal reference band intensity of β-actin or Lamin A/C. Different letters indicate significant differences among treatments (*P* < 0.05). ^*^P < 0.05. Data are mean ± SE (*n* = 5–7)

As shown in Fig. 5, the treatment groups NT-CON, NT-PF and HT-CON had no effect on MMP-9 protein expression, but influenced (*P* < 0.0001) protein expression levels of claudin-1, occludin, JAMA, ZO-1, A20, NF-κB p65 and MMP-2 in the jejunum of broilers on d 42. Compared with the NT-CON, both NT-PF and HT-CON decreased (*P* < 0.05) protein expression levels of claudin-1, occludin, JAMA, ZO-1 and A20, but increased MMP-2 protein expression. Compared with the NT-PT, HT-CON decreased (*P* < 0.05) claudin-1, occludin, JAMA, ZO-1 and A20, and increased NF-κB p65 protein expression. Compared with the HT-CON, all supplemental Zn treatments under HT increased (*P* < 0.01) protein expression levels of claudin-1, occludin, JAMA, ZO-1 and A20, but decreased (*P* < 0.0001) protein expression levels of NF-κB p65 and MMP-2. Under HT, Zn source did not affect protein expression levels of ZO-1, A20, NF-κB p65 and MMP-9, but influenced (*P* < 0.05) protein expression levels of claudin-1, occludin, JAMA and MMP-2. Added Zn level did not affect protein expression levels of ZO-1 and MMP-9, but influenced (*P* < 0.05) protein expression levels of claudin-1, occludin, JAMA, A20, NF-κB p65 and MMP-2*.* No interactions between Zn source and added Zn level were detected on protein expression levels for any of the above-mentioned genes. Compared with the ZnS, Zn-Prot M increased (*P* < 0.05) protein expression levels of claudin-1, occludin and JAMA, but decreased (*P* < 0.05) MMP-2 protein expression. Compared with the 30 mg/kg of supplemental Zn, 60 mg/kg increased (*P* < 0.05) protein expression levels of claudin-1, occludin, JAMA and A20, and decreased (*P* < 0.0001) protein expression levels of NF-κB p65 and MMP-2.

## Discussion

The results from the current study show that HS significantly reduced ADFI, ADG, mRNA expression levels of claudin-1, occludin, *JAMA, ZO-1* and *A20*, and their corresponding protein expression, and remarkably increased F/G, serum FITC-D, endotoxin and IL-1β contents, serum DAO activity as well as MMP-2 activity, *NF-κB p65* mRNA expression level, and protein expression levels of NF-κB p65 and MMP-2 in the jejunum of broilers. However, dietary supplementation of Zn, especially organic Zn as Zn-Prot M at 60 mg/kg, significantly decreased serum FITC-D, endotoxin and IL-1β contents, serum DAO activity as well as MMP-2 activity, *NF-κB p65* mRNA expression level, and protein expression levels of NF-κB p65 and MMP-2, and notably promoted mRNA expression levels of claudin-1, *ZO-1* and *A20*, and protein expression levels of claudin-1, occludin, JAMA, ZO-1 and A20 in the jejunum of HS broilers. The above results suggest that dietary supplemental Zn, especially 60 mg Zn/kg as Zn-Prot M, could alleviate the HS-induced intestinal barrier function damage by promoting the expression of TJ proteins via induction of A20-mediated suppression of the NF-κB p65/MMP-2 pathway (A20/NF-κB p65/MMP-2 pathway) in the jejunum of HS broilers, which supports our hypothesis. These findings have not been reported before, and thus provide a scientific foundation for the alleviation of the HS-induced intestinal barrier function injury through dietary optimal Zn addition in broiler production.

Previous study has indicated that HS has a negative impact on the growth performances of broilers [50]. In the present study, HS decreased ADG and ADFI, and increased F/G of broilers during d 22–42 mainly due to a reduced feed intake, which is consistent with previous findings [51, 52]. Some of the previous studies showed that dietary Zn addition can alleviate HS-induced growth retardation [53, 54]. In other studies, dietary Zn supplementation had no significant effect on the growth performance of broilers exposed to HS [55, 56], which was consistent with our present results. It appears that the effect of dietary Zn on the growth performance of heat-stressed broilers is dependent on the Zn level in basal diets, the dose of supplemental Zn, dietary formulation, and experimental duration time [57, 58].

Earlier studies have demonstrated that HS can impair intestinal barrier function in broilers [59, 60], and that dietary Zn could improve intestinal barrier function [29, 61]. FITC-D, a macromolecular substance, cannot cross the intestinal epithelial barrier under normal conditions [62]. Hence, oral administration of FITC-D has been used as an indicator of intestinal permeability in broilers [63]. Furthermore, when intestinal barrier function is compromised, DAO and endotoxin can be released into blood. Therefore, serum DAO activity and endotoxin content are also regarded as typical indicators for reflecting the intestinal barrier function [64–66]. Alhenaky et al. [59], Tabler et al. [45] and Lan et al. [67] reported that HS-induced damage of intestinal barrier function in broilers is accompanied by an increase in endotoxin and FITC-D contents and DAO activity in serum. Similar results were obtained in the present study. The HS-induced intestinal barrier function damage was caused by the combined effect of both the reduced feed intake and HT itself, but the effect of HT itself was greater than that of the reduced feed intake, which is in line with one previous report [60]. Dietary Zn was shown to alleviate the HS-induced increases in endotoxin content in pig serum [68], FITC-D content in broiler serum [69] and DAO activity in duck serum [70]. Similar results were observed in our study in which dietary Zn supplementation was observed to relieve HS-induced increases in serum FITC-D and endotoxin contents and DAO activity. Furthermore, Zn-Prot M was more effective than ZnS in decreasing the serum endotoxin content of HS broilers, while 60 mg/kg of added Zn was more effective than 30 mg/kg of added Zn in decreasing serum FITC-D and endotoxin contents and DAO activity of broilers, which has not been reported before. Our findings, together with those of others [28, 71, 72], indicate that dietary supplemental Zn, especially 60 mg Zn/kg as Zn-Prot M, can protect the intestinal barrier of broilers from HS-induced damage.

The TJ proteins are pivotal structures for maintaining intestinal integrity and barrier function [73]. Ruff et al. [74] reported that the damage of the small intestine is aggravated with increased HS exposure time. Siddiqui et al. [75] demonstrated that the damage of HS on the jejunum of broilers was more obvious. Therefore, the jejunum of broilers at 42 days of age was selected as the target small intestinal segment in the subsequent measurements of the present study. Alhotan et al. [11] reported that HS damages intestinal barrier function by reducing the expression of claudin-1, occludin and JAMA in the jejunum of broilers. Xia et al. [76] demonstrated that the HS-induced damage of intestinal barrier function is accompanied by decreased mRNA expression levels of claudin-1 and *ZO-1* in pigs. In the present study, we found that HS injures intestinal barrier function partially by down-regulating the expression of claudin-1, occludin, JAMA, and ZO-1 in the jejunum of broilers. Additionally, HS-induced down-regulation in the expression of the above TJ proteins in the jejunum of broilers was attributed to the combined effect of both the reduced feed intake and HT itself, but the effect of HT itself was greater than that of the reduced feed intake. In a study by Xie et al. [77], ZnS supplementation was found to attenuate lipopolysaccharide-induced decreases of claudin-1, occludin and ZO-1 mRNA and protein expression in duck jejunal epithelial cells. Similar results were observed in the current study in which dietary Zn addition relieved HS-induced down-regulation of claudin-1, occludin, JAM-A and ZO-1 protein expression levels in the jejunum of broilers. Moreover, the Zn-Prot M was more effective than ZnS, and 60 mg/kg of supplemental Zn was better than 30 mg/kg. The above results indicate that dietary supplemental Zn, especially 60 mg Zn/kg as Zn-Prot M, can alleviate HS-induced down-regulation of main TJ protein expression in the jejunum of broilers, which has not been reported before. However, the mechanism by which Zn regulates the expression of TJ proteins needs further investigation.

It is reported that the decreased protein expression of TJ proteins is closely correlated with the activation of NF-κB [78]. The NF-κB signaling pathway plays a crucial role in inflammation [79]. The activation of NF-kB promotes the production of inflammatory cytokines TNFα, IL-1β, and IL-6 [80]. In the present study, we found that HS increased the IL-1β content in the serum of broilers mainly due to the effect of HT itself, reflecting NF‐kB activation. A study with bovines indicated that HS-induced decrease in TJ protein expression is associated with NF-κB activation [10]. Furthermore, previous studies revealed that NF-κB is a key transcriptional factor participating in the regulation of MMP-2 and MMP-9 gene expression [81, 82]. Both MMP-2 and MMP-9 are responsible for degrading the intestinal TJ proteins [16, 17]. In the present study, HS increased the jejunal NF-κB p65 and MMP-2 protein expression levels and also MMP-2 activity mainly due to the effect of HT itself. Dietary Zn addition alleviated the HS-induced down-regulation of the jejunal NF-κB p65 mRNA and protein expression levels as well as the level of MMP-2 protein expression and MMP-2 activity. Additionally, the remission effect of Zn-Prot M was greater than that of ZnS, and the remission effect of supplemental Zn was greater at 60 mg/kg than at 30 mg/kg. The above findings suggest that dietary supplemental Zn, especially 60 mg Zn/kg as Zn-Prot M, can alleviate HS-induced down-regulation of main TJ protein expression possibly by inhibiting the NF-κB p65/MMP-2 signaling pathway in the jejunum of broilers, which has not been reported before.

A20 mediates some of the biological actions of Zn [83], and it is required for the Zn-induced NF-κB inactivation [84]. Li et al. [85] reported that dietary Zn supplementation inhibits intestinal inflammation via A20-mediated NF-kB inactivation. In the current investigation, we found that HS markedly decreased *A20* mRNA and its protein expression in the jejunum of broilers mainly due to the effect of HT itself, but dietary Zn addition alleviated the HS-induced down-regulation of the jejunal *A20* mRNA and its protein expression. Moreover, the remission effect of Zn-Prot M was greater than that of ZnS in *A20* mRNA expression, and the remission effect of supplemental Zn was greater at 60 mg/kg than at 30 mg/kg in *A20* mRNA and its protein expressions. Therefore, dietary supplemental Zn, especially 60 mg Zn/kg as Zn-Prot M, can alleviate HS-induced down-regulation of A20 expression in the jejunum of broilers and thereby allow restoration of A20-mediated suppression of the NF-κB p65/MMP-2 pathway (A20/NF-κB p65/MMP-2 pathway), which has not been reported before.

## Conclusions

Dietary supplementation with Zn, especially organic Zn as Zn-Prot M at 60 mg/kg, alleviates HS-induced intestinal barrier function damage by promoting the expression of TJ proteins possibly via induction of A20-mediated suppression of NF-κB p65/MMP-2 pathway (A20/NF-κB p65/MMP-2 pathway) in the jejunum of broilers. Further studies need to be conducted using a primary cultured jejunal epithelial cell model or the jejunal organoid model of broilers as well as gene over-expression and RNA interference to address and confirm the above possible mechanisms.

## Data Availability

The datasets used and/or analyzed during the current study are available from the corresponding author on reasonable request.

## Abbreviations

AA: Arbor Acres
ADFI: Average daily feed intake
ADG: Average daily gain
A20: Zinc finger protein A20
DAO: Diamine oxidase
FITC-D: Fluorescein isothiocyanate dextran
F/G: Feed to gain ratio
HS: Heat stress
IL: Interleukin
JAMA: Junctional adhesion molecule A
MMP: Matrix metalloproteinase
NF-κB p65: Nuclear factor kappa-B p65
TJ: Tight junction protein
ZO-1: Zonula occludens-1
